# Supplementary material for: The Mentor Mothers Program in the Department of Defense in Nigeria: An Evaluation of Healthcare Workers, Mentor Mothers, and Patients’ Experiences
Source: Healthcare (Basel). 2021 Mar 14;9(3):328. doi: 10.3390/healthcare9030328 (PMC8001623; doi:10.3390/healthcare9030328)
Supplement: Supplementary file 1 [file healthcare-09-00328-s001.pdf]

Questionnaire S1: Sample Questionnare

Guided interview schedule.

School of Nursing and Public Health, University of KwaZulu-Natal, Durban-South Africa.

This Questionnaire addressed the Experiences of Healthcare workers, Mentor Mothers & Patients in the Mentor Mothers Program in 4 selected Hospitals namely the Guards Brigade Medical Centre, Nigerian Navy Hospital, Airforce Base Hospital and the Defense Headquarters Medical Centre all in Abuja, Nigeria.

Experiences of Healthcare workers, Mentor Mothers, and patients in the DoD Mentor Mothers' Program.

QUESTIONS FOR HEALTHCARE WORKERS AND MENTOR MOTHERS

Demographic Data (please tick (✓) where applicable).

Age…… 20-30 years

31-40 years

41-50 years

50 years and above

Number of children

•Alive

•Deceased

Level of education

• Secondary school

• Tertiary level

• Post-graduate

• Others…………..(Specify)

Religion

• Christianity

• Islam

• No religion

• Others…………….(specify)

Place of assignment

• Army hospital

• Navy hospital

• Airforce hospital

• DHQ Medical Centre

Duration of employment

- < 6 months
- 6months -5 years
- 5-10 years
- 11-15 years
- 16-20 years
- 20 years & above

Source(s) of income………. (Please specify)……………………….

I would like us to discuss your experience since you commenced working as PMTCT care provider within the Mentor-Mother program in this hospital.

Questions

1.From your perspective, please share with me your views about the MM program

2. Describe your experiences since your deployment in the MM program?

3. In terms of effort, time and commitments, how well have your expectations been met regarding the extent of your involvement in the program ?

4. Was there any advance training conducted / information provided to enable you successfully carry out your duties? If so, name the advance training you participated in.

5. Describe the nature of your training?

6. How useful was the content of the training you received in preparation for the performance of your duties?

7. How timely was the training you received in preparation for the use of the MM program?

8. Describe to me what changes you have observed in service delivery after your training for this program?

9. What aspects of the programme functions well?

10. What aspect of the program is not functioning according to expectation?

11. Comment on the materials you were provided in order to Work with. If materials were lacking, please elaborate.

12. Please describe the nature of communication between you and other stakeholders in the programme.

13. What aspects were challenging with specific stakeholders?

14. How well were your expectations met regarding the frequency and content of information conveyed to you by Management?

15. Overall, comment on the efforts to prepare you for the impact of the mentor-mother program in this hospital?

16. Based on your observation, how well has the Mentor-Mother program met the goals for which it was introduced in the DoD?

17. How does the community surrounding you (your patients inclusive) see the services of the Mentor Mothers? What do you think makes them feel that way?

18. What significant difference have you observed from what obtained previously?

19.What would you say has been responsible?

20.What do you have to say about the utilization of MMs for PMTCT generally?

21. How is fee provided for services rendered, if any?

22. In your opinion what has specifically enabled the successful implementation of the MM program in this hospital?

22. What aspect of the program would you like to be improved upon or discarded?

23. Are there any specific challenges you encounter from your perspective as a provider of services in the programme?

24. From your observation please share if there are any health facility-related factors hindering the implementation of the MM program.

25. If so why do you think they exists?

26. Please describe if there are any community-based factors influencing the implementation of the MM program.

27. How accessible are the ANC/PMTCT services in this hospital?

28. Describe any client-based challenges regarding PMTCT care and services so far.

29. Describe(if any) the logistic challenges regarding the MM program for ANC/PMTCT services experienced so far.

30. Describe the availability of staff with regard to patients' demands and provision of services.

31. Would you say the staff/client ratio is sufficient to cover all aspects of the program?

32. Have you ever referred patients seeking ANC/PMTCT care to other hospitals? If so how often?

33. For what condition did you make the referral ? What were the responses from other health facilities?

34. Are there any aspects of the program that you think have not been properly implemented.

35. What aspect of the program is under or over utilized?

36. What aspect of the program is not functioning according to expectation?

37. What specific support do you need improve the services you provide?

38. Describe if there are any misconceptions about the program so far?

39. Do you have any other general observation or suggestions to make about how the challenges can be addressed?

40.What aspect of the program would you like to be improved upon or discarded?

41. Is there any other aspect we have not mentioned that you would like to elaborate on?

42. If you were given an opportunity to change anything about the programme , what would that be?

43. In conclusion, what is your overall assessment of the outcome of this program?

QUESTIONS FOR PATIENTS

Section one- Demographic Data

Age………… 20-30 years

31-40 years

41-50 years

50 years and above

Number of children…………………………….

- Alive
- Deceased

Level of education

- Secondary school
- Tertiary level
- Post-graduate
- Others…………..(Specify)

Religion

- Christianity
- Islam
- No religion
- Others…………….(specify)

Occupation

- Employed
- Unemployed
- Housewife
- Business

Source(s) of income…….. (please specify)……………………………

Place where registered for ANC/PMTCT care

- Army hospital
- Navy hospital
- Airforce hospital
- DHQ Medical Centre

Experiences of patients in the DoD Mentor Mothers' Program.

Question. ***What have been your experiences as a beneficiary of the mentor-mother program in this hospital?***

Sub questions:

1. Please share how you got to know about the Mentor-Mother program currently running in this hospital?

2. For how long have you been registered for ANC/PMTCT services in this hospital?

3. What is your understanding of the MM program ?

4. How would you describe the Mentor Mothers?

5. In terms of effort, time and commitment, how well have the providers of the MM program met your expectations since you registered in the program?

6. In your own Opinion what is mother-to-child transmission ?

7. If not addressed, what do you think could happen to the unborn child?

8. Describe your experiences while accessing ANC/PMTCT care in this hospital

9. How well have your expectations been met after registration in the ANC/PMTCT program in this hospital(e.g., effort, time, commitments, etc.)?

10. Comment on the quality of services you are receiving in this hospital?

11. What specific support do you receive while accessing PMTCT services in this hospital

12. How effective was this support ?

13. What aspects did you find helpful with this program?

14. What aspect of the program has been not so helpful to you since you started attending clinic in this hospital?.

15 From your perspective What aspects of the program functions well?

16 What aspect of the program is not functioning according to your expectation?

17. What aspect would you recommend be changed?

18. Are there any practical suggestions you have, if so what are they?

19. What can you identity as the outcome of this program?

20. If you had to make any changes to the overall programme what will that be in terms of priority?

21. In your opinion what has been the acceptability of the MM program in this hospital?

22. . What changes have you observed in service delivery after you enrolled for this program?

23. From your perspective, what do you think has been responsible for this changes?

24. Describe the nature of communication between you and the providers of this programme?

25. If you had to improve the communication between you and the service providers in this project, what would you suggest?.

26. Which particular stakeholder would you suggest improving communication with ?

27. How well were your expectations met regarding the frequency and content of information conveyed to you by Management?

28. Elaborate on some of the expectations that were not met?

29. What would be your suggestions for the improvement about this?

30. Comment on any meetings you have participated in for this programme?

31. What information did you receive?

32. How has that information impacted you subsequently?

33. What other information would you suggest be included in the program If at all?

34.What are some of the factors that have enabled you to remain enrolled in the program?

35. As recipients of the service of m2m can you identify any practical challenges you have encountered in the program?

36. Specify any challenges you have encountered doctors,

37. Specify any challenges you have encountered nurses,

38 .Specify any challenges you have had with the Mentor Mothers

39. Have you been involved in evaluation or assessment of the programme? If so how many of these evaluations/ assessments have you participated in ?

40. Over-all how would you rate the Mentor-Mother Program in this hospital?

41. What advice do you have for those engaging with PMTCT/ANC services in this hospital?

42. What else would you like to share about the prevention of mother-to-child transmission of HIV?
